# Stable maintenance of the Mre11-Rad50-Nbs1 complex is sufficient to restore the DNA double-strand break response in cells lacking RecQL4 helicase activity

**DOI:** 10.1016/j.jbc.2021.101148

**Published:** 2021-08-30

**Authors:** Hyunsup Kim, Hyemin Choi, Jun-Sub Im, Soon-Young Park, Gwangsu Shin, Jung-Ho Yoo, Gyungmin Kim, Joon-Kyu Lee

**Affiliations:** 1 Interdisciplinary Graduate Program in Genetic Engineering, Seoul National University, Seoul, Korea; 2 Department of Biology Education, Seoul National University, Seoul, Korea

**Keywords:** RecQL4, DNA double-strand break, the Mre11-Rad50-Nbs1 complex, Usp28, Rothmund-Thomson syndrome, γH2AX, phosphorylated H2AX, ATM, Ataxia telangiectasia mutated, CtIP, CtBP-interacting protein, DSB, double-strand break, EGFP, enhanced green fluorescent protein, HR, homologous recombination, MRN, Mre11-Rad50-Nbs1, NCS, neocarzinostatin, NHEJ, nonhomologous end joining, RTS, Rothmund–Thomson syndrome

## Abstract

The proper cellular response to DNA double-strand breaks (DSBs) is critical for maintaining the integrity of the genome. RecQL4, a DNA helicase of which mutations are associated with Rothmund–Thomson syndrome (RTS), is required for the DNA DSB response. However, the mechanism by which RecQL4 performs these essential roles in the DSB response remains unknown. Here, we show that RecQL4 and its helicase activity are required for maintaining the stability of the Mre11-Rad50-Nbs1 (MRN) complex on DSB sites during a DSB response. We found using immunocytochemistry and live-cell imaging that the MRN complex is prematurely disassembled from DSB sites in a manner dependent upon Skp2-mediated ubiquitination of Nbs1 in RecQL4-defective cells. This early disassembly of the MRN complex could be prevented by altering the ubiquitination site of Nbs1 or by expressing a deubiquitinase, Usp28, which sufficiently restored homologous recombination repair and ATM, a major checkpoint kinase against DNA DSBs, activation abilities in RTS, and RecQL4-depleted cells. These results suggest that the essential role of RecQL4 in the DSB response is to maintain the stability of the MRN complex on DSB sites and that defects in the DSB response in cells of patients with RTS can be recovered by controlling the stability of the MRN complex.

Proper cellular response to DNA double-strand breaks (DSBs), the most cytotoxic type of DNA damage, is critical for maintaining the integrity of genome to avoid instability that may result in genetic diseases, carcinogenesis, and cell death (1, 2). In response to DSBs, cells activate a checkpoint kinase, ataxia telangiectasia mutated (ATM), which is involved in the control of cell cycle progression and recovery from DNA damage (3). Subsequently, DSBs are generally repaired by nonhomologous end joining (NHEJ) or homologous recombination (HR) (4).

In both DNA DSB repair and checkpoint activation processes, the Mre11-Rad50-Nbs1 (MRN) complex plays essential roles as a sensor, a signal transducer, and an effector for DSB response (5). The MRN complex recognizes and binds to DSB sites and then recruits and activates ATM, a central regulator of the DSB response signaling pathway (6). ATM phosphorylates histone H2AX at DSB sites, and this phosphorylated H2AX (γH2AX) is recognized by MDC1, which mediates a feed-forward loop that promotes activation of ATM and γH2AX in a large domain near the damaged DNA sites (7). Other checkpoint mediators, such as 53BP1 and Brca1, key factors for DSB repair pathway choice, are also recruited to MDC1 and contribute to ATM activation and further repair events (8).

The MRN complex also plays an essential role in the DNA end resection process during HR repair (9). After the binding of the MRN complex on the DSB sites, Mre11 initiates short-range resection of DSB ends with CtBP-interacting protein (CtIP); subsequently, an extensive 3′-end protruding single-stranded DNA is generated by the action of ExoI and Dna2 (10, 11). Following the resection, single-stranded DNA binding protein, RPA, is replaced by Rad51, which induces the homology search and DNA strand invasion for HR (12, 13).

Another important player for DNA DSB responses is the RecQ family helicase (14, 15). These helicases are well conserved from prokaryote to eukaryote, and humans have five RecQ helicases: RecQL1, WRN, BLM, RecQL4, and RecQL5 (15). These helicases play various roles in maintaining genome integrity, and their mutations are associated with genetic diseases. Defects in BLM, WRN, and RecQL4 are associated with Bloom, Werner, and Rothmund–Thomson syndromes (RTS), all of which present genomic instability and cancer disposition (14). Patients with Werner syndrome typically have an aging appearance and early onset of age-related disorders after adolescence (16). RTS presents pleotropic phenotypes, including poikiloderma and cancer predisposition (17), and Bloom syndrome commonly features short stature, sun-sensitive skin, growth deficiency, and immune abnormalities (18).

WRN, BLM, and RecQL4 are all involved in the DNA DSB repair process in various ways, and their helicase activity is crucial for several processes. WRN stimulates NHEJ by its helicase and exonuclease activities (19). BLM plays both pro- and anti-recombination roles by stimulating the end resection activity of Dna2 and by the displacement of Rad51 from resected DNA intermediates (20). RecQL4 has been shown to be recruited very rapidly to DSB sites (21), and its helicase activity is reportedly required for both HR repair (22, 23) and activation of ATM (24) in response to DNA DSBs. RecQL4 has also been postulated as a participant in 5′-end resection by Mre11 and CtIP during HR repair (23); however, how the protein plays a role in this process is not known.

In this study, we examined DSB response in RecQL4-defective cells and found that the MRN complex is prematurely disassembled from DSB sites. Furthermore, preventing its early disassembly from DSBs is sufficient to restore DSB response in RecQL4-defective cells. Our results showed that the essential role of RecQL4 in DSB response is the stable maintenance of the MRN complex and defects in DSB response in RTS cells can be recovered by counteracting the ubiquitination of Nbs1 through expression of Usp28.

## Results

### The MRN complex is prematurely disassembled from DSB sites in RTS and RecQL4-depleted cells

**Figure 1:**
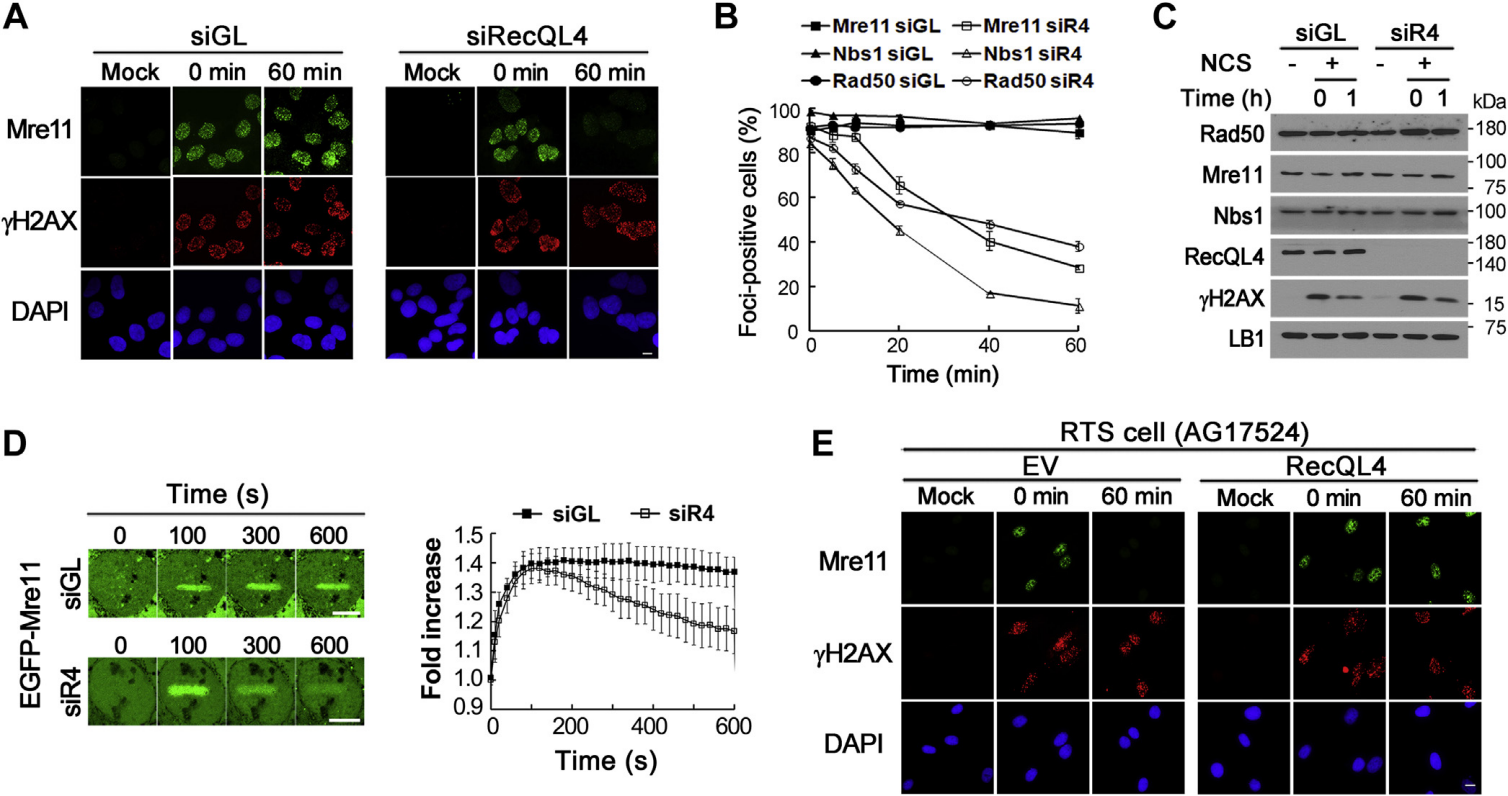
**The MRN complex is prematurely disassembled from double-strand break sites in RecQL4-defective cells.***A* and *B*, immunostaining of the MRN component proteins. U2OS cells transfected with RecQL4 or GL2 (siGL) siRNAs were treated with neocarzinostatin for 15 min and incubated in a fresh medium for the indicated times. Representative images of Mre11 immunostaining (*A*) and graphs for the percentages of foci-positive cells (*B*) are shown. Data in graphs are means ± SD; n = 3. The scale bar represents 10 μm. *C*, Western blots of U2OS cells prepared as in (*A*) and (*B*). *D*, EGFP-Mre11 binding to microirradiation sites in RecQL4- (siR4) or mock-depleted (siGL) U2OS cells. Data in graphs are means ± SD; n = 25. The scale bar represents 10 μm. *E*, immunostaining of Mre11 in Rothmund–Thomson syndrome (RTS) cells (AG17524) transfected with empty (EV) or RecQL4 expression vectors. Cells were treated with neocarzinostatin for 15 min and incubated in a fresh medium for the indicated times. The scale bar represents 10 μm.

Because both HR repair and ATM activation were reported to be impaired in RecQL4-defective cells (22, 23, 24), we reasoned that factors commonly involved in both these processes may be affected by the defects in RecQL4. By analyzing proteins at DSB sites using immunostaining after treating cells with neocarzinostatin (NCS), a DSB-inducing reagent, we found that MRN foci formed in RecQL4-depleted U2OS cells, as in mock-depleted cells, but they started to rapidly disappear during the postincubation period (Fig. 1, *A* and *B*). Foci of all three MRN component proteins were reduced, but Nbs1 foci decreased most rapidly, and the proportion of Nbs1 foci-positive cells was less than 20% after incubation for 40 min (Fig. 1*B*). In contrast, total cellular levels of the MRN component proteins did not change significantly (Fig. 1*C*), suggesting that depletion of RecQL4 may affect the MRN complex assembled on DSB sites only and results in premature disassembly of the MRN complex from these sites. Early dissociation of the MRN complex from DSB sites was also observed in RecQL4-depleted cells subjected to laser microirradiation (Fig. 1*D*). The EGFP-fused Mre11 proteins rapidly bound to the microirradiation site, and their binding was found to be dependent on Nbs1 (Fig. S1), suggesting that the binding of EGFP-fused Mre11 represented binding of the MRN complex to microirradiation sites. The level of EGFP-fused Mre11 bound to microirradiation sites reached a peak at around 100 s after microirradiation and then decreased in RecQL4-depleted cells, whereas this level did not change significantly in mock-depleted cells (Fig. 1*D*). Premature disassembly of the MRN complex from DSB sites, which was prevented by expression of RecQL4, was also observed in NCS-treated fibroblast cells collected from patients with RTS (Fig. 1*E* and Fig. S2).

### The helicase activity of RecQL4 is required for stable maintenance of the MRN complex on DSB sites during DSB response

**Figure 2:**
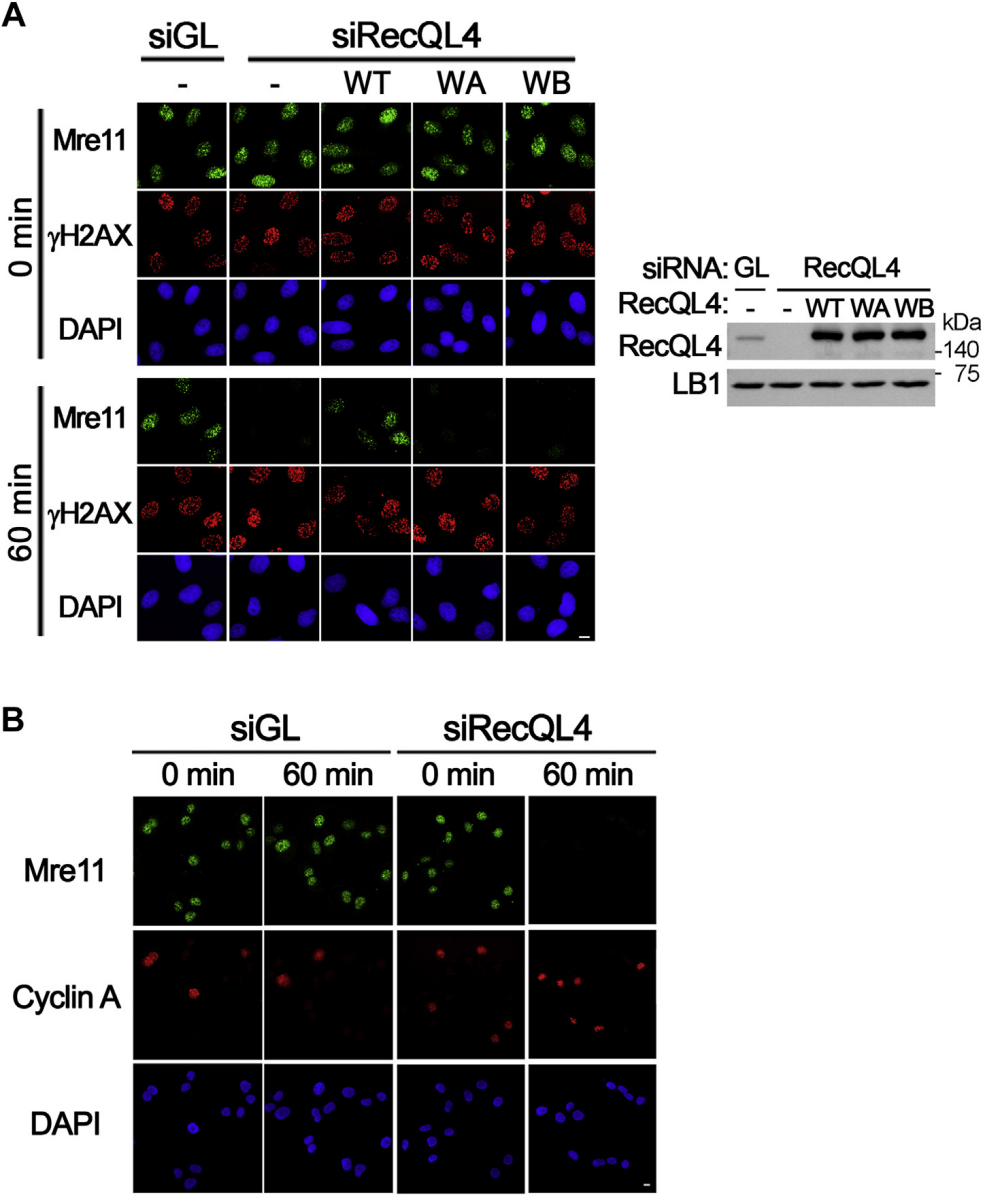
**Stable maintenance of the MRN complex on double-strand break sites requires helicase activity of RecQL4.***A*, immunostaining of RecQL4-depleted U2OS cells transfected with wildtype (WT), Walker A (WA), or Walker B (WB) mutant RecQL4. Cells were treated with neocarzinostatin and then incubated in fresh medium for the indicated times. *Right panel* is the Western blot showing depletion of endogenous RecQL4 and expression of wildtype and mutant RecQL4 proteins. *B*, immunostaining of Mre11 and Cyclin A in mock- (siGL) or RecQL4-depleted U2OS cells treated with neocarzinostatin and released for 0 or 1 h. The scale bar represents 10 μm.

To test whether the helicase activity of RecQL4 contributes to the stable maintenance of the MRN complex during DSB response, we expressed RecQL4 mutant proteins defective in ATP binding (K508G in Walker A motif) or ATP hydrolysis (D605A and E606A in Walker B motif) in RecQL4-depleted cells. Subsequently, we examined Mre11 foci after NCS treatment. Early disappearance of Mre11 foci was prevented by the expression of wildtype RecQL4 proteins, whereas Mre11 foci still disappeared during incubation in cells expressing mutant RecQL4 proteins defective in DNA helicase activity (Fig. 2*A*). As the N terminus of RecQL4 is essential for DNA replication, depletion of RecQL4 might indirectly affect the stability of MRN complexes on DSB sites by altering the cell cycle. However, premature disassembly of the MRN complex was observed in both cyclin A–positive and –negative cells (Fig. 2*B*), which ruled out the possibility that depletion of RecQL4 might indirectly affect the MRN stability by altering the cell cycle. Furthermore, expression of Walker A or Walker B mutant RecQL4 proteins, which contain intact N termini, thereby supporting DNA replication, failed to restore the stability of the MRN complex on DSB sites (Fig. 2*A*). Collectively, these results suggest that the MRN complex formed on DSB sites is prematurely disassembled in RecQL4-defective cells owing to the lack of RecQL4 helicase activity.

### Skp2-dependent ubiquitination is responsible for premature disassembly of the MRN complex from DSB sites

**Figure 3:**
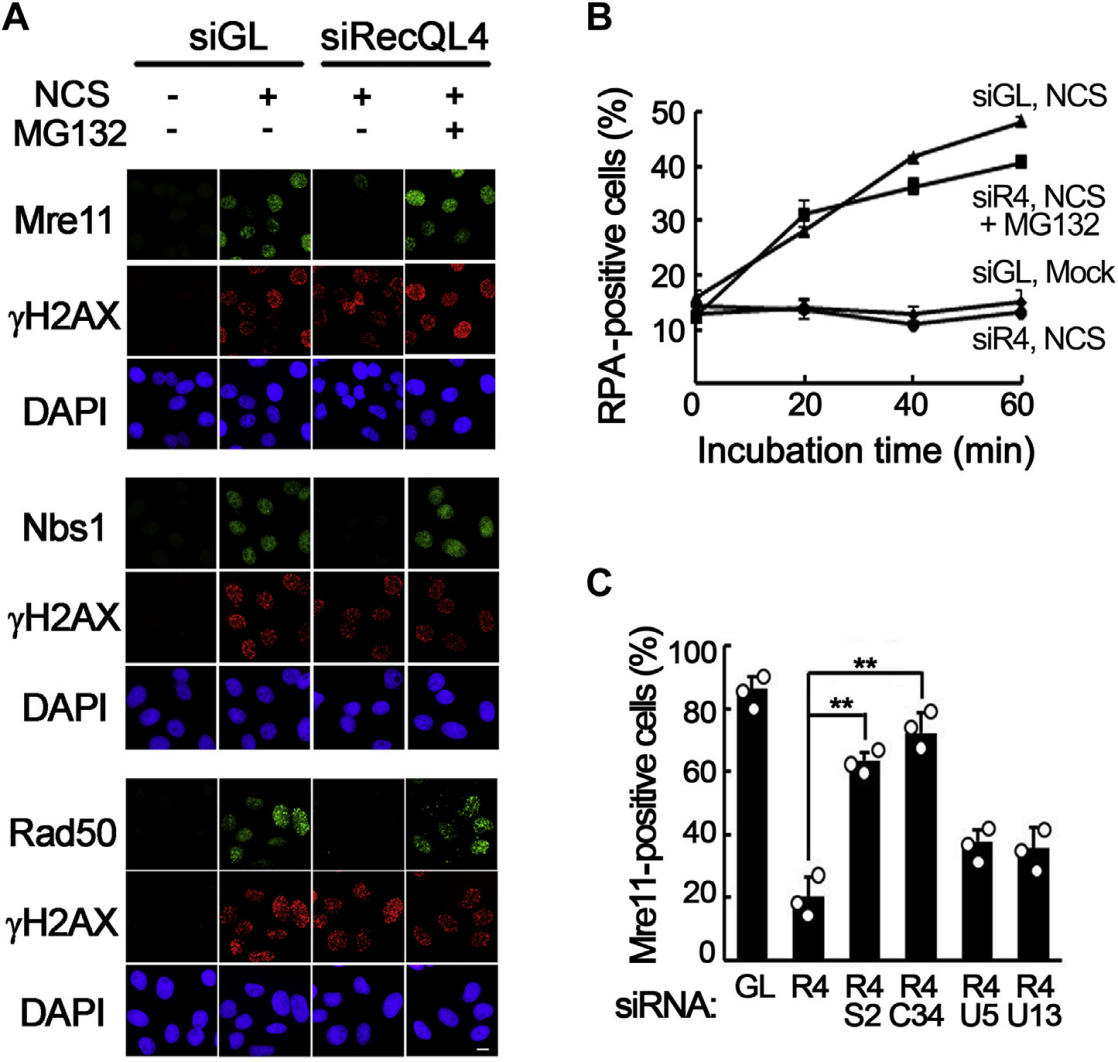
**Skp2-dependent ubiquitination is responsible for premature disassembly of the MRN complex.***A*, immunostaining of Mre11, Nbs1, or Rad50 in mock- (siGL) or RecQL4-depleted U2OS cells treated with neocarzinostatin (NCS) and released for 1 h in the absence (−) or presence (+) of MG132 (50 μg/ml). The scale bar represents 10 μm. *B*, percentages of RPA foci-positive cells in mock- or RecQL4-depleted (siR4) U2OS cells after mock or NCS treatment in the absence or presence of MG132. Data in graphs are means ± SD; n = 3. *C*, percentages of Mre11 foci-positive cells, 1 h after NCS treatment, among cells depleted for the indicated proteins. R4, RecQL4; S2, Skp2; C34, Cdc34; U5, Ubc5; U13, Ubc13. Data in graphs are means ± SD; n = 3. ∗∗*p* < 0.01.

To obtain an insight into how the MRN complex is prematurely disassembled from DSB sites, we examined whether the kinase associated with DSB response or proteasome is required for this process. Although inhibitors of ATM or DNA-dependent protein kinase did not prevent premature disassembly of the MRN complex (Fig. S3), MRN foci were stably maintained in RecQL4-depleted cells treated with a proteasome inhibitor, MG132 (Fig. 3*A*), suggesting that disassembly of the MRN complex from DSB sites may be an active process requiring proteasomal activity. Furthermore, inhibition of the proteasome not only prevented premature disassembly of the MRN complex but also restored the DNA end resection activity for DSB repair, which was judged by the appearance of foci for RPA2, a component of single-stranded DNA binding protein (Fig. 3*B*). Therefore, premature disassembly of the MRN complex from DSB sites may be responsible for the known defects in DSB response found in RecQL4-defective cells.

Given that, among the three MRN proteins, Nbs1 appeared to be removed from DSB sites most rapidly, we explored the possibility that Nbs1 may be a target for ubiquitination. Previously, Nbs1 was shown to be ubiquitinated by Skp2 E3 ligase (25). Therefore, we first examined whether Skp2 E3 ligase is involved in the instability of the MRN complex in RecQL4-depleted cells. Depletion of Skp2 or Cdc34, an E2 ubiquitin-conjugating enzyme supporting lysine (K) 48-linked ubiquitination (26), clearly increased stability of the MRN complex on DSB sites, as judged by the appearance of Mre11 foci in RecQL4-depleted cells, whereas depletion of Ubc5 or Ubc13, two other E2 ubiquitin-conjugating enzymes supporting K48- or K63-linked ubiquitination, did not (Fig. 3*C*). These results suggest that Skp2-E3 ligase, using Cdc34 as an E2, may be responsible for the instability of the MRN complex observed in RecQL4-defective cells.

### K48-linked ubiquitination of Nbs1 by Skp2 E3 ligase increases in cells with DSBs

**Figure 4:**
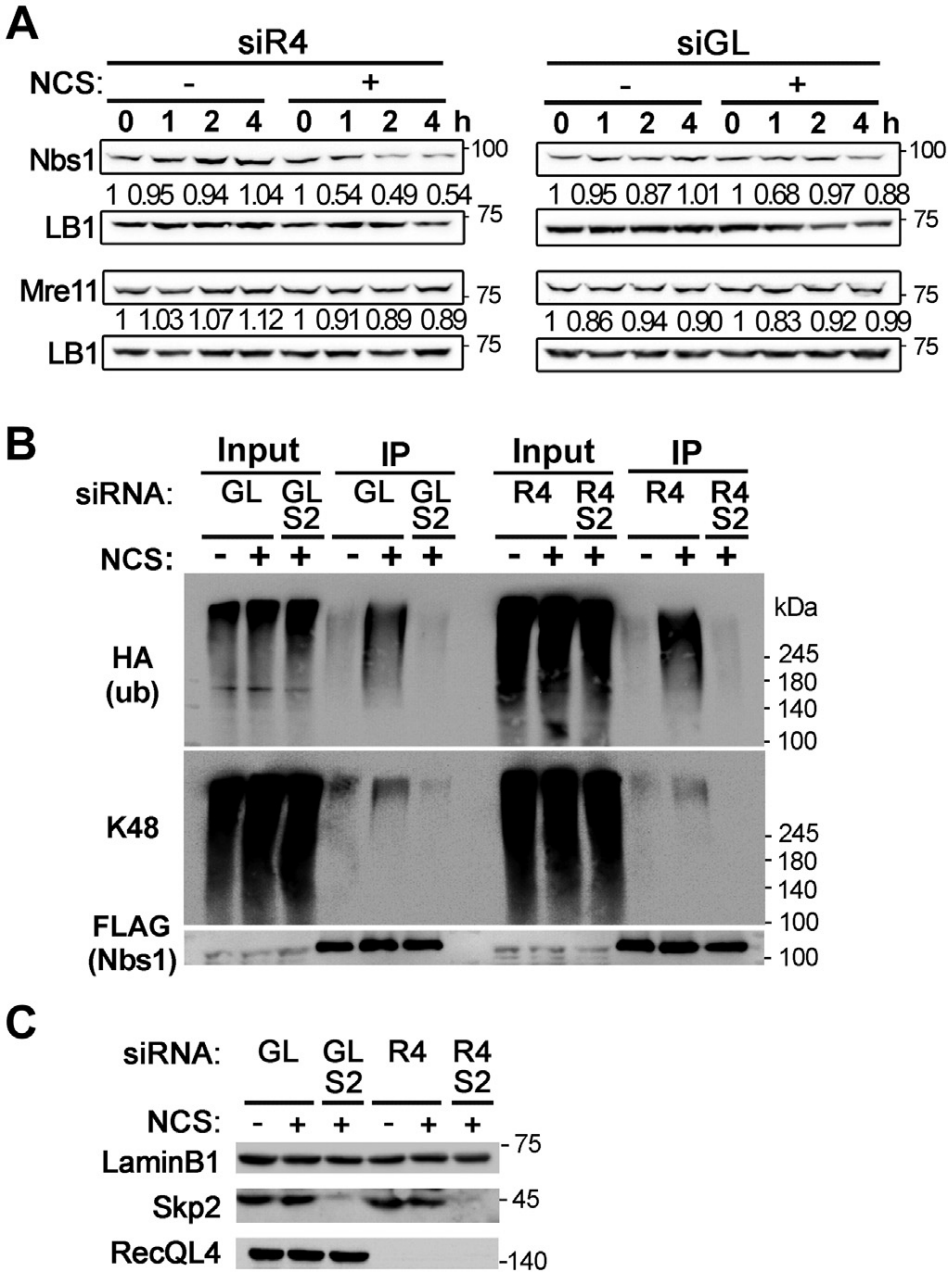
**Skp2-dependent ubiquitination of Nbs1 occurs in cells treated with NCS.***A*, stability of Nbs1 and Mre11 proteins in RecQL4- or mock-depleted U2OS cells treated with NCS for indicated times. All cells were treated with 50 μg/ml cycloheximide. The levels of Mre11 and Nbs1 proteins normalized to that of Lamin B1 (LB) from three independent experiments are shown below each lane. *B*, ubiquitination of Nbs1 in 293T cells depleted of RecQL4 and/or Skp2, 1 h after NCS or mock treatment. Nbs1 proteins were immunoprecipitated with anti-FLAG antibody, and anti-HA (for ubiquitin), anti-K48 ubiquitin (K48), and anti-FLAG (for Nbs1) antibodies were used for Western blotting. *C*, Western blots showing depletion of Skp2 and RecQL4 in cells used in (*B*). NCS, neocarzinostatin.

As Skp2-E3 ligase, using Cdc34 as an E2, supports K48-linked ubiquitination that ultimately results in proteasome-dependent degradation, we examined whether degradation of the MRN component proteins occurs in RecQL4-depleted cells during DSB response. We did not detect a significant decrease in the total protein level of any MRN component proteins in RecQL4-depleted cells after NCS treatment (Fig. 1*C*). However, the stability of the Nbs1 protein, which was observed in cells treated with cycloheximide, decreased in RecQL4-depleted cells in a DSB-dependent manner, whereas the stability of Mre11, another MRN component protein, did not change significantly (Fig. 4*A*). Furthermore, K48-linked ubiquitination of Nbs1 was significantly increased in both mock- and RecQL4-depleted cells after NCS treatment and depletion of Skp2 almost completely eliminated this ubiquitination (Fig. 4, *B* and *C*). Therefore, K48-linked ubiquitination of Nbs1 by Skp2 E3 ligase indeed occurs in cells treated with NCS and appears to be responsible for the premature disassembly of the MRN complex from DSB sites in RecQL4-depleted cells.

### Stable maintenance of the MRN complex is sufficient to restore the DSB response in RecQL4-defective cells

**Figure 5:**
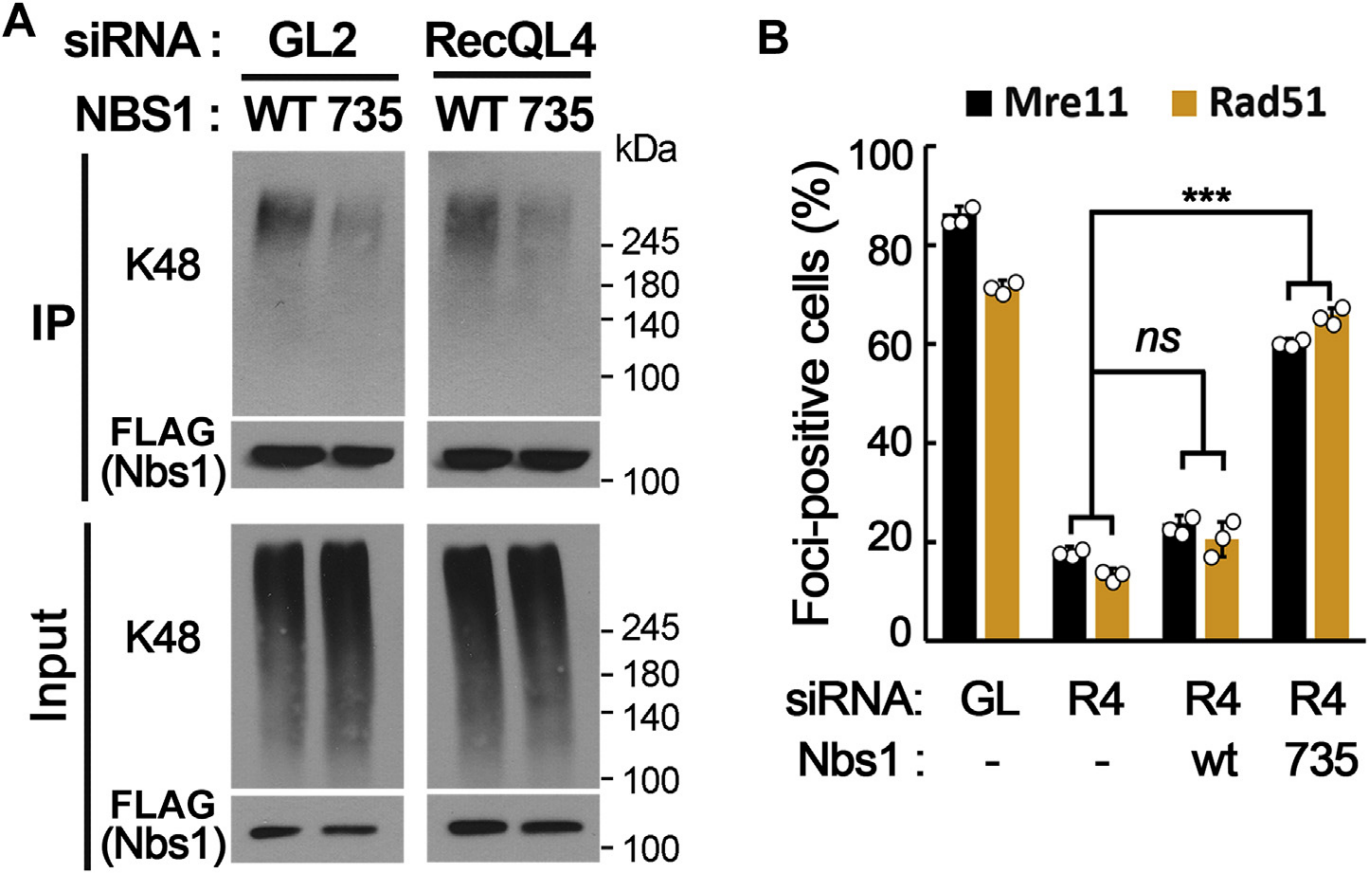
**Defects in double-strand break response are restored by preventing Skp2-dependent ubiquitination of Nbs1 in RecQL4-defective cells.***A*, ubiquitination of wildtype (WT) or K735R Nbs1 (735) in mock- or RecQL4-depleted 293T cells treated with neocarzinostatin. *B*, percentages of Mre11 and Rad51 foci-positive cells, 1 h after neocarzinostatin treatment in cells expressing wildtype (WT) or K735R (735) Nbs1. ∗∗∗*p* < 0.001; *ns*, not significant.

If ubiquitination of Nbs1 and premature disassembly of the MRN complex from DSB sites are responsible for the known defects in RecQL4-defective cells, preventing their ubiquitination or degradation would be sufficient to restore the DSB response. To test this possibility, we expressed Nbs1 mutant proteins containing a lysine-to-arginine substitution (K735R) at the site of Skp2-dependent ubiquitination (25) and examined whether the stability of the MRN complex in DSB sites and HR repair ability were affected in RecQL4-depleted cells. Although lysine at amino acid residue 735 (K735) was mapped as a major K63-ubiquitination site by Skp2 E3 ligase in a previous study (25), K48-linked ubiquitination was also significantly reduced in K735R Nbs1 compared with wildtype Nbs1 (Fig. 5*A*), suggesting that K735 is a major K48-ubiquitination site in Nbs1. Expression of K735R Nbs1 in RecQL4-depleted cells clearly increased stability of the MRN complex and HR repair ability, as judged by the appearance of Mre11 and Rad51 foci after NCS treatment, whereas the expression of wildtype Nbs1 did not (Fig. 5*B*). These results suggested that defects in MRN stability and HR repair ability in RecQL4-defective cells can be recovered by preventing Skp2-dependent ubiquitination of the Nbs1.

**Figure 6:**
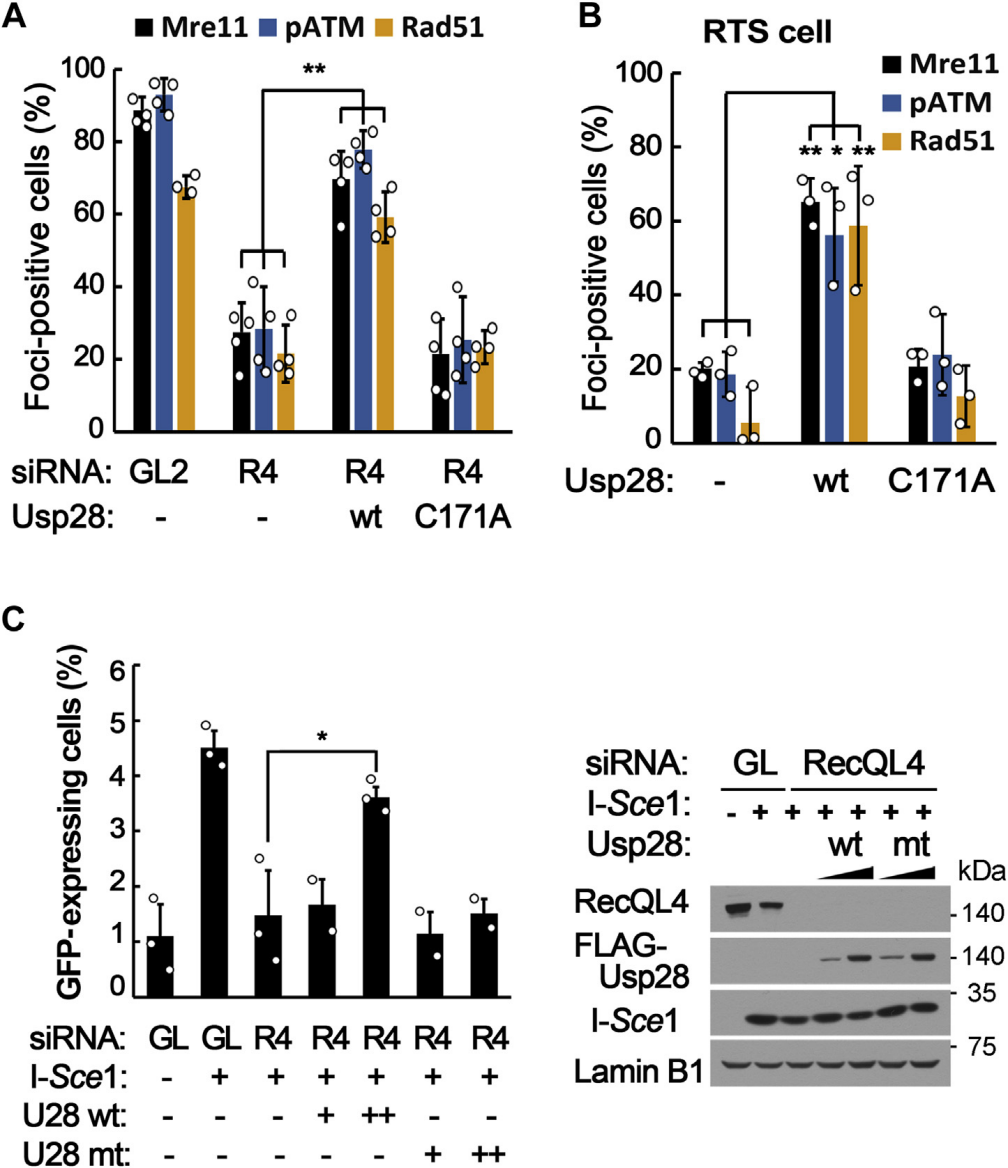
**Defects in double-strand break response are restored by the expression of Usp28 in RTS and RecQL4-depleted cells.***A* and *B*, percentages of Mre11-, phospho-ATM-, or Rad51 foci-positive cells after neocarzinostatin treatment in RecQL4-depleted U2OS cells (*A*) and RTS (AG17524) cells (*B*) transfected with empty (−), wildtype Usp28 (wt), or C171A mutant Usp28 plasmids. *C*, HR repair assay using DR-GPF-integrated U2OS cells. Mock (GL) or RecQL4 (R4)-depleted cells were transfected with I-*Sce*I and WT or C171A mutant Usp28 plasmids. *Right panel* is the Western blots showing the expression levels of RecQL4, Usp28, and I-*Sce*I. Data in graphs are means ± SD; n = 4 (for *A*) or 3 (for *B* and *C*). ∗∗*p* < 0.01, ∗*p* < 0.05.

Another possible way to prevent ubiquitin-dependent degradation would be the overexpression of a deubiquitinase that can act on the target protein. Therefore, we explored deubiquitinases that could prevent premature disassembly of the MRN complex after overexpression in RecQL4-defective cells. After testing several deubiquitinases that were shown to be associated with DNA metabolism, we found that Usp28 overexpression resulted in the stable maintenance of the MRN complex on DSB sites in RTS and RecQL4-depleted U2OS cells, whereas overexpression of a catalytically inactive Usp28 (C171A) did not (Fig. 6, *A* and *B* and Fig. S4). Furthermore, formation of phospho-ATM and Rad51 foci, which are markers of ATM activation and HR repair, respectively, was significantly increased by the overexpression of Usp28 (Fig. 6, *A* and *B*). We also found that Usp28 overexpression in RecQL4-depleted DR-GFP HR reporter cells and in RTS cells increased the HR repair ability (Fig. 6*C*) and resistance to DSB-inducing reagents (Fig. S5), respectively. Therefore, Usp28 overexpression almost completely restored ATM activation and HR repair abilities in RecQL4-defective cells.

### Usp28 counteracts the ubiquitination of Nbs1 to stabilize the MRN complex on DSBs and to restore DSB response in RecQL4-defective cells

**Figure 7:**
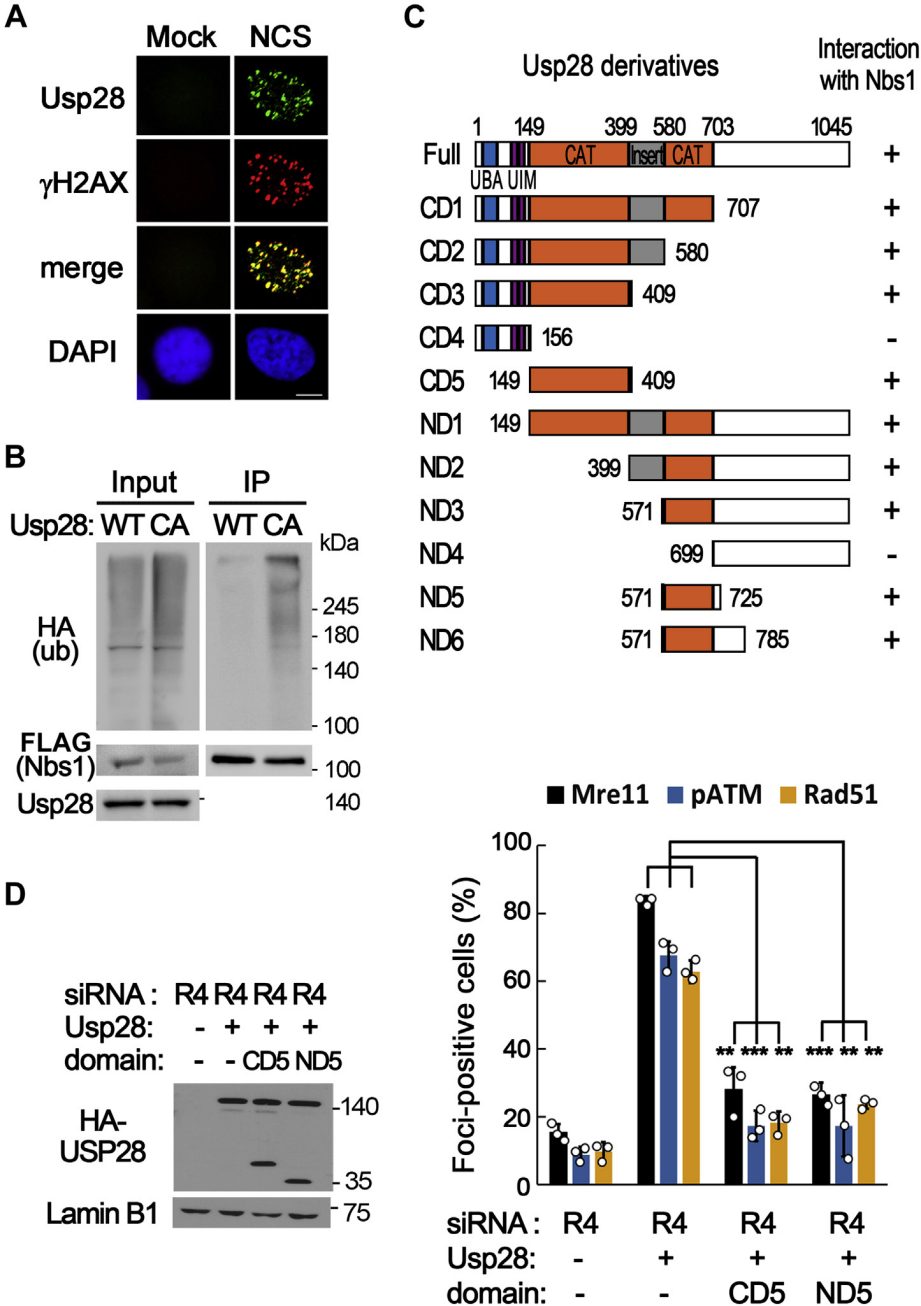
**Usp28 counteracts the ubiquitination of Nbs1 to stabilize the MRN complex and to restore double-strand break (DSB) response in RecQL4-depleted cells.***A*, localization of Usp28 at DSB sites in U2OS cells, 1 h after neocarzinostatin (NCS) or mock treatment. The scale bar represents 10 μm. *B*, ubiquitination of Nbs1 in cells expressing wt or C171A mutant Usp28, 1 h after NCS treatment. *C*, schematic diagram of truncated Usp28 proteins and their interaction with Nbs1. *D*, expression of Nbs1 interaction domains in Usp28 inhibits the restoration of DSB responses by Usp28 overexpression. Percentages of Mre11-, phospho-ATM-, or Rad51 foci-positive cells transfected with the indicated proteins. Data in graphs are means ± SD; n = 3. ∗∗∗*p* < 0.001, ∗∗*p* < 0.01. CAT, catalytic domain; UBA, ubiquitin-associated domain; UIM, ubiquitin-interaction motif.

Usp28 protein is localized in DSB sites after NCS treatment (Fig. 7*A*) and interacted directly with Nbs1 proteins in coimmunoprecipitation experiments (Fig. 7*C*). Furthermore, ubiquitination of Nbs1 is significantly reduced by overexpression of wildtype Usp28 but not by catalytically inactive Usp28 (Fig. 7*B*). Therefore, overexpression of Usp28 appeared to restore MRN stability on DSB sites in RecQL4-defective cells by directly acting on the Nbs1 protein. To further confirm this notion, we determined the domain of Usp28 responsible for interacting with Nbs1 and examined the effect of its overexpression on the stability of the MRN complex and DSB response. There are two Nbs1-interacting domains in Usp28 (Fig. 7*C* and Fig. S6), and coexpression of any one of these domains (amino acids 149–409 or 571–725) as competitors prevented the recovery of MRN stability, ATM activation, and HR repair by the overexpression of Usp28 in RecQL4-depleted cells (Fig. 7*D*). This result suggested that the overexpression of Usp28 restored the DSB response in RecQL4-defective cells by directly acting on the MRN complex.

## Discussion

All RecQ helicases in mammalian cells have been shown to be involved in DNA DSB responses (14, 15), and direct participation of WRN and BLM have been well documented in many studies; WRN stimulates NHEJ by its helicase and exonuclease activities (19), and BLM plays both pro- and anti-recombination roles by stimulating the end resection activity of Dna2 (27) and by the displacement of Rad51 from resected DNA intermediates (20). As RecQL4 has a conserved helicase domain, and its helicase activity is required for end resection by Mre11-CtIP during HR repair, RecQL4 has also been postulated as participating in the end resection process (23). In this study, however, we demonstrate that the end resection process occurs in RecQL4-defective cells as long as the stability of the MRN complex is maintained either by mutation in ubiquitination sites on Nbs1 (Fig. 5) or expression of Usp28 (Fig. 6). Therefore, it is evident that RecQL4 does not directly participate in the HR repair process as a component of the end resection complex.

Ubiquitination of proteins at DSB sites has been extensively studied and shown to play important roles in DSB response. These roles include promoting the recruitment of checkpoint and repair proteins at damage sites, altering protein–protein interactions, and clearing repair signaling (28, 29). In this study, we observed K48-linked ubiquitination of Nbs1, which appeared to be responsible for premature disassembly of the MRN complex from DSB sites in RecQL4-defective cells. Ubiquitination of Nbs1 was previously observed in several studies. RNF8-dependent ubiquitination of Nbs1 is reportedly important for optimal binding of Nbs1 to DSB sites (30), and Nbs1 ubiquitination by Skp2 E3 ligase or Pellino1 plays an important role in ATM activation (25, 31). However, those reports were on K6- or K63-linked ubiquitination, which are known to affect protein–protein interaction. Therefore, the current study is the first to reveal the K48-linked ubiquitination of Nbs1. Although the reported ubiquitination of Nbs1 by Skp2 E3 ligase was a K63-linked form (25), Skp2 E3 ligase targets many proteins, including p27, for degradation by K48-linked ubiquitination (32, 33). Consistent with this notion, K48-linked and Skp2-dependent ubiquitination of Nbs1 was observed in NCS-treated cells (Fig. 4*B*), and depletion of Ubc13, which was shown to be an E2 for K63-ubiquitination of Nbs1 (25), did not prevent premature disassembly of the MRN complex in RecQL4-depleted cells, whereas depletion of Cdc34, which supports K48-linked ubiquitination (26), did (Fig. 3*C*). These results strongly suggest that K-48 linked ubiquitination of Nbs1 indeed occurs and is responsible for premature disassembly of the MRN complex from DSB sites in RecQL4-defective cells.

Of interest, instability of Nbs1 proteins and premature disassembly of the MRN complex from DSB sites were observed only in RecQL4-depleted cells (Figs. 1 and 4*A*), although K48-linked ubiquitination of Nbs1 occurred in both mock- and RecQL4-depleted cells (Fig. 4*B*). In addition, interaction of Skp2 with the MRN complex was observed regardless of the presence of RecQL4 proteins (Fig. S7). Therefore, the absence of RecQL4 may not influence the stability of the MRN complex *via* affecting the ubiquitination step. There must be a mechanism to control the stability of the MRN complex during DSB response, and RecQL4 appears to be essential for this control. As overexpression of Usp28 stabilizes the MRN complex, deubiquitination may be a plausible mechanism for stabilization of the MRN complex. Usp28 was initially reported to play an important role in DSB response by stabilizing many proteins, including 53BP1, Chk2, Mdc1, and Nbs1, in H460 lung carcinoma cells (34). However, unlike H460 cells, Usp28 depletion did not decrease the stability of these proteins in other cell lines (34) and had a minor effect on the DNA repair process in U2OS cells (35). Therefore, results of these studies imply the existence of other players that are defective in H460 cells and influence the stability of proteins involved in DSB response. Since many deubiquitinases are recruited to DSB sites, where they play essential roles, other deubiquitinases may also play a redundant role to stabilize the MRN complex during DSB response.

Although we have unequivocally demonstrated the essential role of RecQL4 in stable maintenance of the MRN complex during DSB response, we still do not understand how RecQL4 and its helicase activity influence the stability of the MRN complex during DSB response. It may be possible that RecQL4 is involved in the stabilization process or in the recruitment of a factor or factors that play a role in the stabilization of the MRN complex, such as deubiquitinases. Previously, RecQL4 proteins were shown to bind rapidly to laser microirradiation sites, and Mre11 and its nuclease activity were found to be required for the maintenance of RecQL4 proteins on the microirradiation site (23). However, in our hands, RecQL4 proteins are rapidly recruited to the microirradiation site but stay there only transiently (peaks around 100 s) (Fig. S8*A*). Moreover, depletion of Mre11 or inhibition of its nuclease activity by mirin treatment does not significantly affect the association and dissociation patterns of RecQL4 (Fig. S8*B*). These observations are further supported by a study on the protein dynamics in DNA lesions (21). In this study, cell lines expressing GFP-fused repair proteins under the control of their own promoters were examined, and RecQL4 was shown to bind to microirradiation site faster than Rad50, a component of the MRN complex, and begin to dissociate at the time of Rad50 binding. Therefore, we assume that RecQL4 cannot directly participate in the stabilization or recruitment processes throughout the DSB response. As RecQL4 has intrinsic DNA helicase activity, it has potential to influence the binding or removal of proteins around DSB sties. Therefore, RecQL4 and its helicase activity may indirectly play a role in MRN stabilization by influencing the binding and/or modifications of proteins around DSB sites during the short stay of RecQL4 at the DSB sites. However, we still cannot rule out the possibility that a small amount of RecQL4 protein remains at the DSB site and participates directly in the stabilization process throughout the DSB response.

Importance of RecQL4 helicase activity in DSB response has been demonstrated both here and in previous studies, which all used human culture cells (23, 24, 36). However, this notion was challenged by a recent knock-in mouse study. Knock-in mice with an amino acid substitution at the ATP binding motif in the DNA helicase domain of RecQL4 appeared normal in development, hematopoiesis, and B and T cell development. Furthermore, a cell line with this mutant allele was as sensitive to several DNA damage-inducing reagents as the isogenic control cell line (37), suggesting that the helicase activity of RecQL4 may not be required for DNA repair. Although we still do not clearly understand what causes differences between mouse and human cells, our results in this study indicate that defects in DSB response caused by loss of RecQL4 could be complemented by other activities. Different expression levels of deubiquitinases targeting the MRN complex or any differences in the control mechanism governing the stability of the MRN complex between mouse and human systems may influence cellular response to DSBs. Whether premature disassembly of the MRN complex is caused by defects in RecQL4 and is prevented by expressing deubiquitinases in mouse cells remain to be seen.

Although type II RTS is caused by mutations in a single gene, RecQL4, patients with RTS showed pleiotropic phenotypes, and clinical features are extremely heterogeneous. All patients have a characteristic skin rash called poikiloderma and many or a few other features including sparse hair, small stature, dental and nail abnormalities, skeletal abnormalities, and increased risk of cancer (17). As RecQL4 has been implicated in many cellular functions (15, 38), multiple and heterogeneous phenotypes may be attributed to multiple functions of RecQL4 and the position of mutations in each patient with RTS. However, it is still hard to understand why the majority of the symptoms of RTS are concentrated in certain tissues such as skin and bones, despite RecQL4 playing roles in basic functions required for the maintenance of any cell. We still do not clearly understand what functions of RecQL4 are responsible for each symptom of patients with RTS. However, if any of the clinical features of RTS is caused by defects in HR or DSB response, differences in the expression level of deubiqutinases in different tissues or cells may give a clue to answer this question. In addition, it would be interesting to determine whether the expression of Usp28 in these tissues reduces the known symptoms of patients with RTS or RTS model animals.

## Experimental procedures

### Cell culture and reagents

U2OS, U2OS harboring DR-GFP reporter for HR assay, and HEK293T cells were cultured in Dulbecco's modified Eagle's medium, supplemented with 10% fetal bovine serum and antibiotics. RTS skin primary fibroblasts cells (AG17524 and AG18371) were obtained from Coriell cell repositories and cultured in alpha Minimum Essential Medium Eagle, supplemented with 15% fetal bovine serum and antibiotics. All the cells were maintained at 37 °C in a humidified incubator containing 5% CO_2_. Transfection of plasmids was performed with Lipofectamine 3000 (Invitrogen). For depletion of proteins, siRNAs were transfected with an electroporator (Invitrogen) and incubated for 2 days. All siRNAs were synthesized by ST Pharm. The sequences of the sense strand of siRNAs used in this study were as follows: GL2 (targeting firefly luciferase), 5′-AACGUACGCGGAAUACUUCGA-3′; RecQL4, 5′-GACUGAGGACCUGGGCAAA-3′; Skp2, 5′-GAUAGUGUCAUGCUAAAGAAU-3′; Cdc34, 5′-GGAAGUGGAAAGAGAGAGCAA-3′; Ubc5, 5′-GAGAAUGGACUCAGAAAUA-3′; Ubc13, 5′-CCAGAUGAUCCAUUAGCAA-3′; Usp28, 5′-CUGCAUUCACCUUAUCAUU-3′. For generating DNA DSBs, cells were treated with NCS (N9162, Sigma-Aldrich) at a concentration of 200 ng/ml for 15 min, unless indicated otherwise. Proteasome inhibitor, MG132, was purchased from Apexbio (A2585). ATM inhibitor, KU-55933 (SML1109), was from Sigma-Aldrich. DNA-PK inhibitor, NU7441 (Axon 1463) was from Axon Medchem (Axon 2678). For the expression of mutant RecQL4 defective in helicase activity, cDNAs encoding RecQL4 with amino acid substitution in Walker A motif (K508G) or Walker B motif (D605A and E606A) were generated by PCR and subcloned into pcDNA3.1(−) plasmid. For the expression of Usp28 derivatives, cDNAs encoding Usp28 fragments were generated by PCR and individually subcloned into pcDNA 3.1(−) plasmid tagged with HA and SV40 nuclear localization signal.

### Antibodies

Primary antibodies used in this study were as follows (Table S1): Anti-RecQL4 antibody was prepared by Abfrontier by immunizing rabbits with recombinant N-terminal (amino acid residues 1–241) RecQL4. Other antibodies used in this study were as follows: Anti-pATM (Ser-1981, #4526, Cell Signaling), anti-Mre11 (GTX30294, GeneTex), anti-Nbs1 (A7703, ABclonal), anti-Rad50 (sc-74460, Santa Cruz), anti-Rad51 (GTX100469, GeneTex), anti-RPA32 (MABE-286, EMD Millipore), anti-Usp28 (A9292, ABclonal), anti-Skp2 (sc-7164, Santa Cruz), anti-γH2AX (A300-081A, Bethyl and 05-636, EMD Millipore), anti-lamin B1 (ab16048, Abcam), anti-GAPDH (sc25778, Santa Cruz), anti-FLAG M2 (Sigma), and anti-HA (AE008, ABclonal). The following antibodies were used as secondary antibodies in immunofluorescence microscopy: Alexa Fluor 488 anti-mouse IgG (A11001, Thermo Fisher), Alexa Fluor 488 anti-rabbit IgG (A11008, Thermo Fisher), Alexa Fluor 594 anti-mouse IgG (A11005, Thermo Fisher), and Alexa Fluor 594 anti-rabbit IgG (A11012, Thermo Fisher).

### Immunofluorescence and laser microirradiation

For immunostaining of Mre11, Nbs1, Rad50, Rad51, RPA32, and Usp28, cells grown on coverslips were pretreated with a buffer containing nonionic detergent (10 mM Pipes pH 7.0, 100 mM NaCl, 300 mM sucrose, 3 mM MgCl_2_, 0.5% Triton X-100) on ice for 5 min and fixed with 4% paraformaldehyde in PBS for 10 min at 25 °C. Fixed cells were washed with PBS containing 0.25% Triton X-100 on ice for 10 min and then incubated in a blocking buffer (5% BSA and 0.25% Triton X-100 in PBS) for 30 min at 25 °C. The indicated proteins were labeled with the respective primary and secondary antibodies in the blocking buffer for 1 h at 25 °C. The nuclei of cells were stained with 0.1 μg/ml 4′-6′-diamidino-2-phenylindole (DAPI) in PBS for 3 min in the dark, and the stained cells were imaged by fluorescent microscopy (Nikon TE2000-U or Zeiss LSM880). For quantitation, cells containing 20 or more foci were considered as foci-positive cells.

For laser microirradiation, U2OS cells grown in glass-bottomed dishes (SPL, 101350) were treated with 5 μg/ml Hoechst 33342 10 min prior to microirradiation. An LSM880 laser confocal microscope system with a temperature-controlled CO_2_ chamber (Zeiss) was used. Fixed wavelength of laser (405 nm) at a scan speed of 32.77 μs/pixel with five iterations and Plan-Apochromat 63X oil objective lens were used. Defined regions of interest were irradiated with 20% laser output. Time-lapse images were captured and fluorescence intensities of irradiated areas relative to those of nonirradiated areas within the nucleus were obtained using the ZEISS ZEN 2.3 SP1 software (Zeiss).

### Immunoprecipitation and immunoblotting

For immunoprecipitation, cells were lysed in a buffer containing 40 mM Tris-HCl, pH 7.5, 100 mM NaCl, 2.5 mM MgCl_2_, 1 mM dithiothreitol, 5% glycerol, 0.2% NP-40, 20 mM NaF, 0.1 mM sodium orthovanadate, and protease inhibitors. After sonication, benzonase (90 units/ml) was added and the reaction was incubated at 4 °C for 4 h. Cell lysates were cleared by centrifugation at 18,000*g* for 10 min and used for immunoprecipitation. To prepare whole-cell extracts for immunoblotting, cells were lysed in a buffer containing 40 mM Tris-HCl, pH 7.5, 150 mM NaCl, 1% NP-40, 1 mM ethylenediaminetetraacetic acid (EDTA), 0.25% sodium deoxycholate, 20 mM NaF, 0.1 mM sodium orthovanadate, and protease inhibitors. The cells were disrupted with sonication, and the concentrations of proteins were measured by Bradford assay. Approximately 30 μg of protein was subjected to sodium dodecyl sulfate–polyacrylamide gel electrophoresis.

### Ubiquitination assay

Ubiquitination assay for Nbs1 ubiquitination was performed as described by Choo and Zhang (39) with slight modification. HEK293T cells were transfected with expression vectors for HA-tagged ubiquitin and indicated proteins and incubated for 24 h. Thereafter, the cells were pretreated with MG132 (40 μM) for 1 h, following NCS treatment (200 ng/ml). Protein extracts were prepared by boiling the cells in cell lysis buffer (20 mM Tris-HCl, pH 8.0, 150 mM NaCl, 2% SDS, 10 mM N-ethylmaleimide and protease inhibitors) for 10 min and then shearing by sonication. The extracts were diluted with nine times the volume of dilution buffer (10 mM Tris-HCl, pH 8.0, 150 mM NaCl, 2 mM EDTA, 1% Triton X-100) and incubated at 4 °C for 30 min with occasional mixing by rotation. The cell extracts were cleared by centrifugation at 18,000*g* for 30 min, and the supernatant was used for immunoprecipitation. Beads were washed twice with immunoprecipitation buffer and three times with wash buffer (10 mM Tris-HCl, pH 8.0, 1 M NaCl, 1 mM EDTA, 1% NP-40).

### Protein stability assay

U2OS cells were transfected with the indicated siRNAs and incubated for 48 h. Before harvest, the cells were pretreated with 50 μg/ml cycloheximide (01810, Sigma) for 1 h and then treated with NCS (200 ng/ml). The cells were harvested and lysed at the indicated times, and the levels of Mre11 and Nbs1 proteins were quantified with a chemiluminescence imaging system (ATTO Ez-CaptureII, CS analyzer) after immunoblotting.

### Homologous recombination repair assay

U2OS cells carrying the HR reporter, DR-GFP, were obtained from Dr Jeremy M. Stark (City of Hope Medical Center). The stable pDR-GFP cells transfected with control or RecQL4 siRNAs were further transfected with RecQL4, USP28 wildtype, or mutant expressing vector with I-*Sce*I-expressing vector. After incubation for 48 h, the GPF-positive cells were analyzed using a flow cytometer (BD, Accuri) with the FlowJo_V.10.1 software.

### WST-1 assay for cell viability

The assay was carried out as described in the manufacturer instruction (EZ-Cytox, DoGenBio). In brief, AG18371 cells transfected with RecQL4, wildtype Usp28, or C171A mutant Usp28 were cultured in 96-well plates and exposed to different concentrations of bleomycin (sc-200134a, Santa Cruz) or cisplatin (NB2251, Novus). After incubation for 48 h, cells were treated with 10% WST-1 reagent (EZ-Cytox, DoGEN) for 1 h, and absorbance was measured using Epoch2 Microplate Spectrophotometer (BioTek).

### Statistical analysis

Statistical significance between groups was determined by two-tailed Student’s *t* test using GraphPad Prism5 software. Data are presented as mean ± SD. All of the statistical tests performed are indicated in the figure legends.

## Data availability

All data are contained within the article and the Supporting Information.

## Supporting information

This article contains supporting information (24).

## Conflict of interest

The authors declare that they have no conflicts of interest with the contents of this article.
