# Supplementary material for: Stable maintenance of the Mre11-Rad50-Nbs1 complex is sufficient to restore the DNA double-strand break response in cells lacking RecQL4 helicase activity
Source: J Biol Chem. 2021 Aug 30;297(4):101148. doi: 10.1016/j.jbc.2021.101148 (PMC8495703; doi:10.1016/j.jbc.2021.101148)
Supplement: Supplemental Figures S1–S8 and Table S1 [file mmc1.docx]

**Supplementary information**

Stable maintenance of the Mre11-Rad50-Nbs1 complex is sufficient to restore the DNA double-strand break response in cells lacking RecQL4 helicase activity

Hyunsup Kim^1§^, Hyemin Choi^2§^, Jun-Sub Im^2^, Soon-Young Park^2^, Gwangsu Shin^1^, Jung-Ho Yoo^2^, Gyungmin Kim^2^, and Joon-Kyu Lee^1,2*^

Figure S1, Figure S2, Figure S3, Figure S4, Figure S5, Figure S6, Figure S7, Table S1


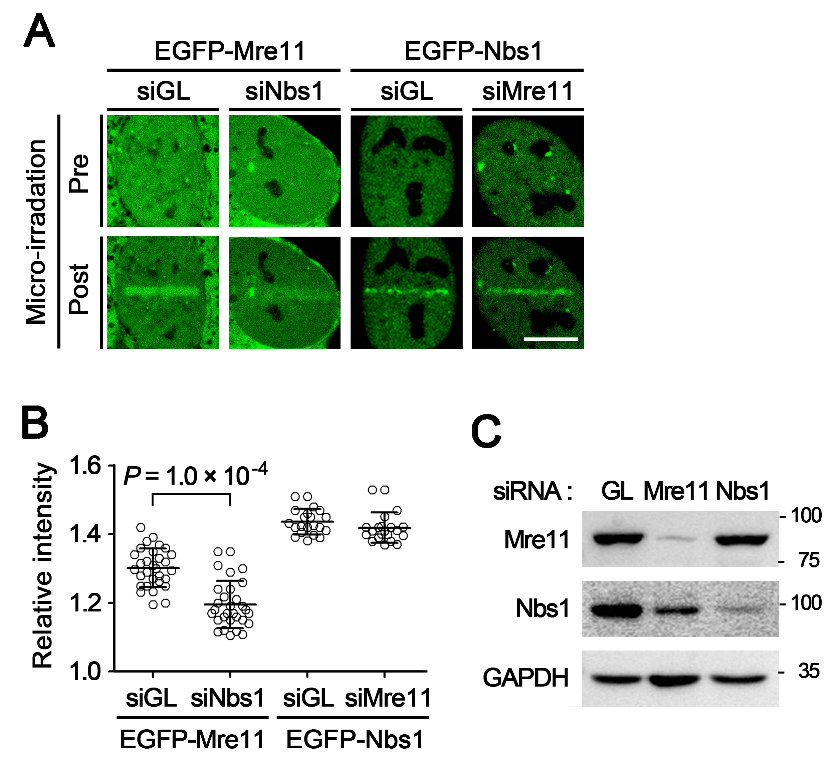


**Figure S1. Recruitment of Mre11 to the micro-irradiated site is dependent upon Nbs1, whereas the recruitment of Nbs1 is not dependent upon Mre11.** Binding of EGFP-Mre11 and EGFP-Nbs1 to laser micro-irradiated sites was observed in cells transfected with the indicated siRNAs and EGFP fusion protein expression vectors, 100 s after micro-irradiation. *A,* Representative images of EGFP-Mre11 and EGFP-Nbs1 binding to DNA damage sites. Scale bar: 10 µm. *B,* Dot plots of relative fluorescence intensity in mock- (siGL), Nbs1- (siNbs1), or Mre11- (siMre11) depleted U2OS cells expressing EGFP-Mre11 or EGFP-Nbs1. Black lines are means ± s.d.; n = 30 (for EGFP-Mre11) and 20 (for EGFP-Nbs1). *C,* Western blot analysis showing depletion of Mre11 and Nbs1.


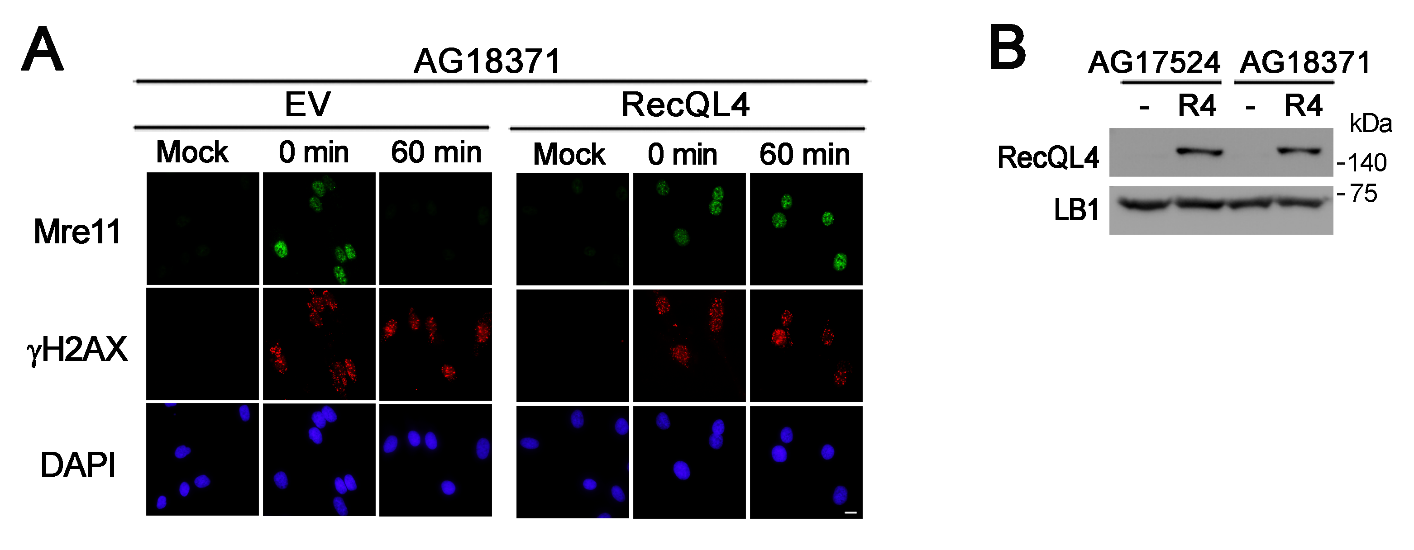


**Figure S2. Instability of the MRN complex in RTS cells is recovered by expression of RecQL4.** *A,* Immunostaining of Mre11 in RTS cells (AG18371) transfected with empty (EV) or RecQL4 expression vectors. Cells were treated with neocarzinostatin (NCS; 200 ng/mL) for 15 min and incubated in a fresh medium for the indicated times. Scale bar: 10 µm. *B,* Western blots of RTS cells prepared as in Fig.1E and Fig.S2A.


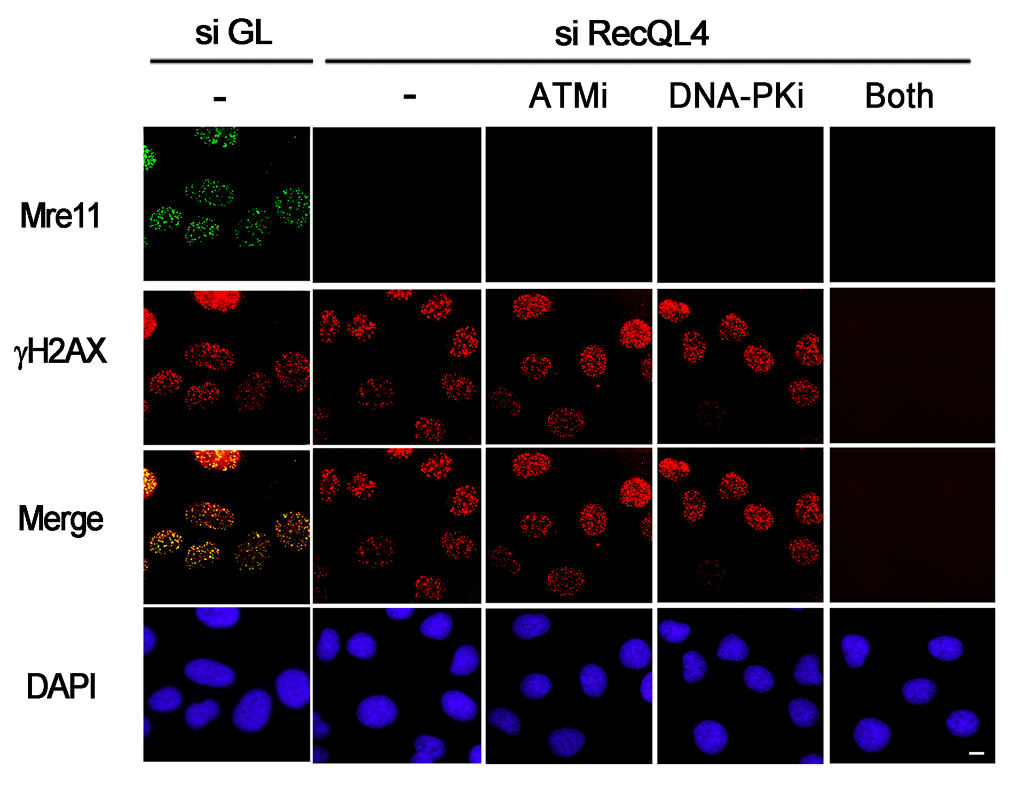


**Figure S3. Inhibition of double-strand break (DSB)-dependent kinases did not prevent premature disassembly of the Mre11-Rad50-Nbs1 complex from DSBs.** GL2 (siGL)- or RecQL4 (siRecQL4)-depleted U2OS cells were treated with 200 ng/mL neocarzinostatin (NCS) for 15 min and incubated for 1 h in the presence or absence of an ATM (10 μM Ku55933) and/or DNA-PK (10 μM NU7441) inhibitor. Immunofluorescence staining was performed with anti-Mre11 and anti-γH2AX antibodies. Cells were treated with the inhibitors, 1 h before the NCS treatment. Scale bar: 10 µm.


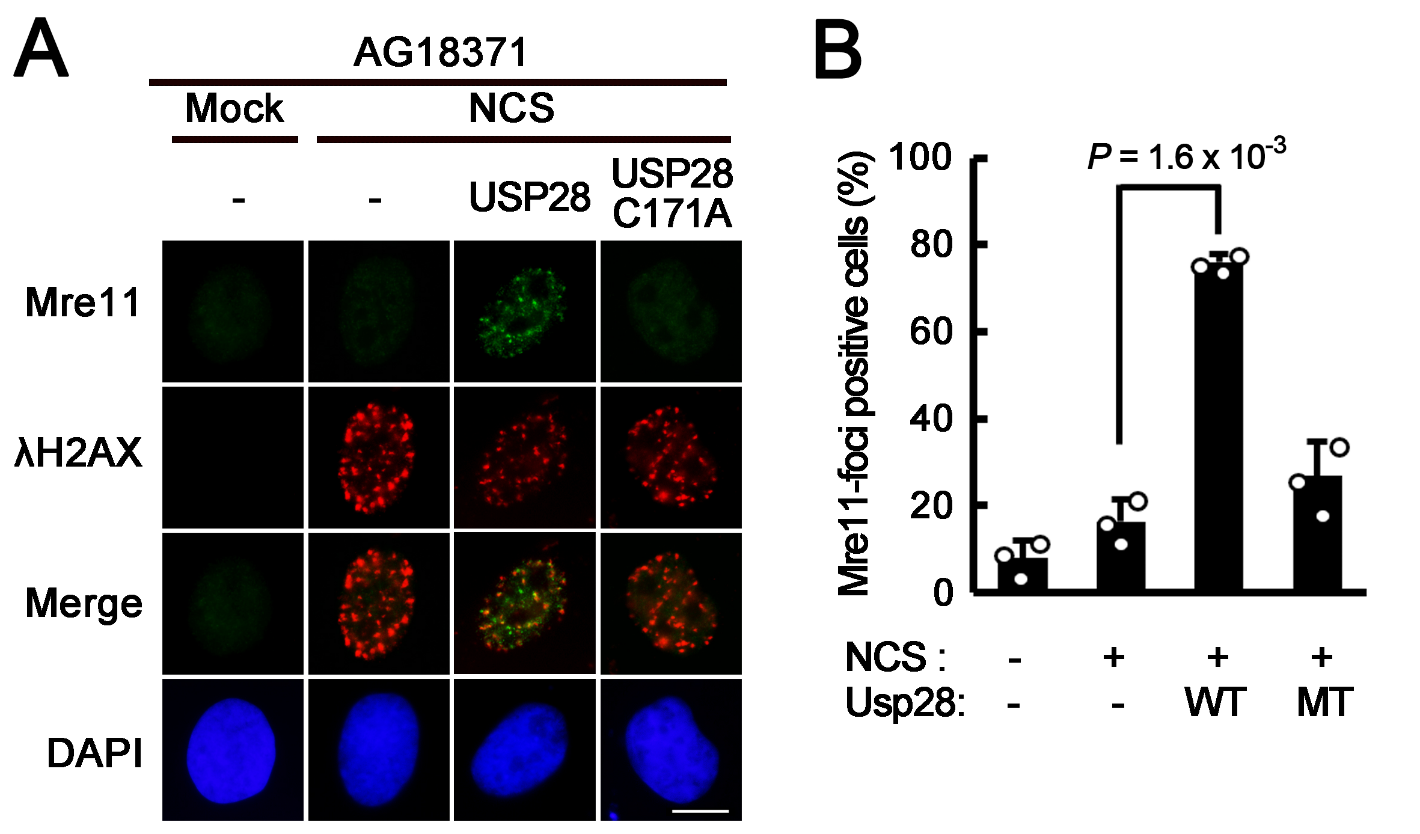


**Figure S4. Stability of the Mre11-Rad50-Nbs1 complex on double-strand break sites is restored in Rothmund-Thomson syndrome (RTS) cells by overexpression of Usp28.** RTS AG18371 cells transfected with plasmids expressing Usp28 WT, C171A mutant, or empty vector were treated with neocarzinostatin (NCS; 200 ng/mL) for 15 min and incubated in fresh medium for 1 h. Immunofluorescence staining was performed, and representative images (**A**) and percentage of foci-positive cells are shown (**B**). Scale bar: 10 µm. Data are means ± s.d.; n = 3.


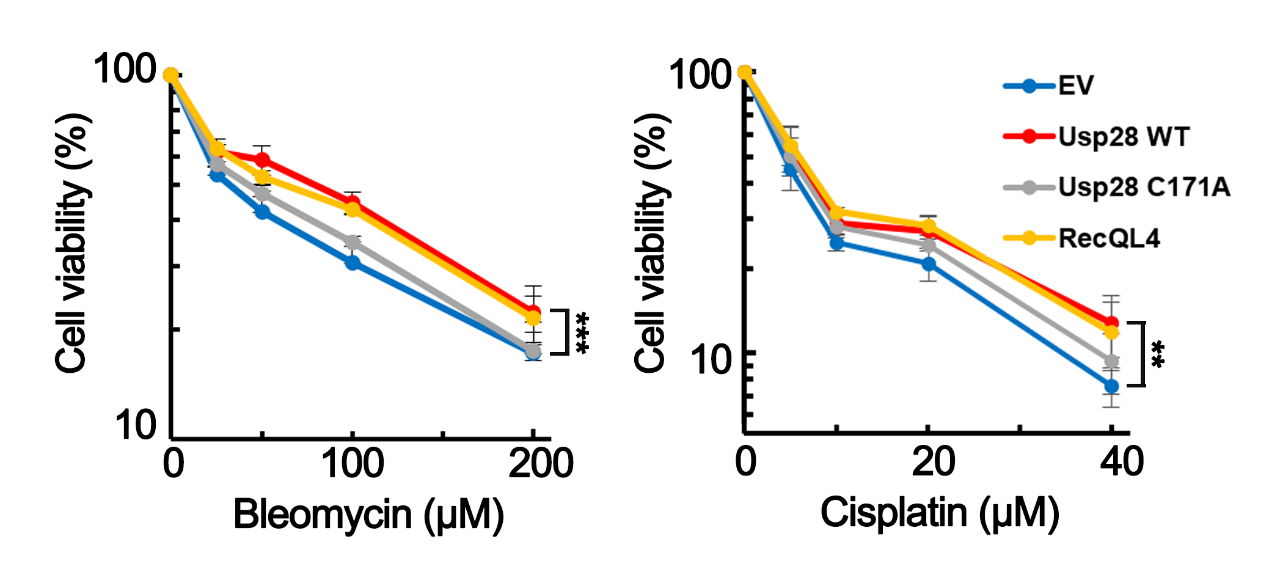


**Figure S5. Expression of Usp28 as well as RecQL4 decreases sensitivity of RTS cells to DSB inducing reagents.** RTS (AG18371) cells transfected with empty, wild type Usp28, C171A mutant Usp28 or RecQL4 plasmids were treated with various concentration of bleomycin or cisplatin for 48 h. WST-1 assay was carried out to measure the percentage of viable cells relative to undamaged cells. Data in graphs are means ± s.e.m.; n = 4. P values for Usp28 wt and empty vector control were presented. *** *P* < 0.001, ** *P* < 0.01.


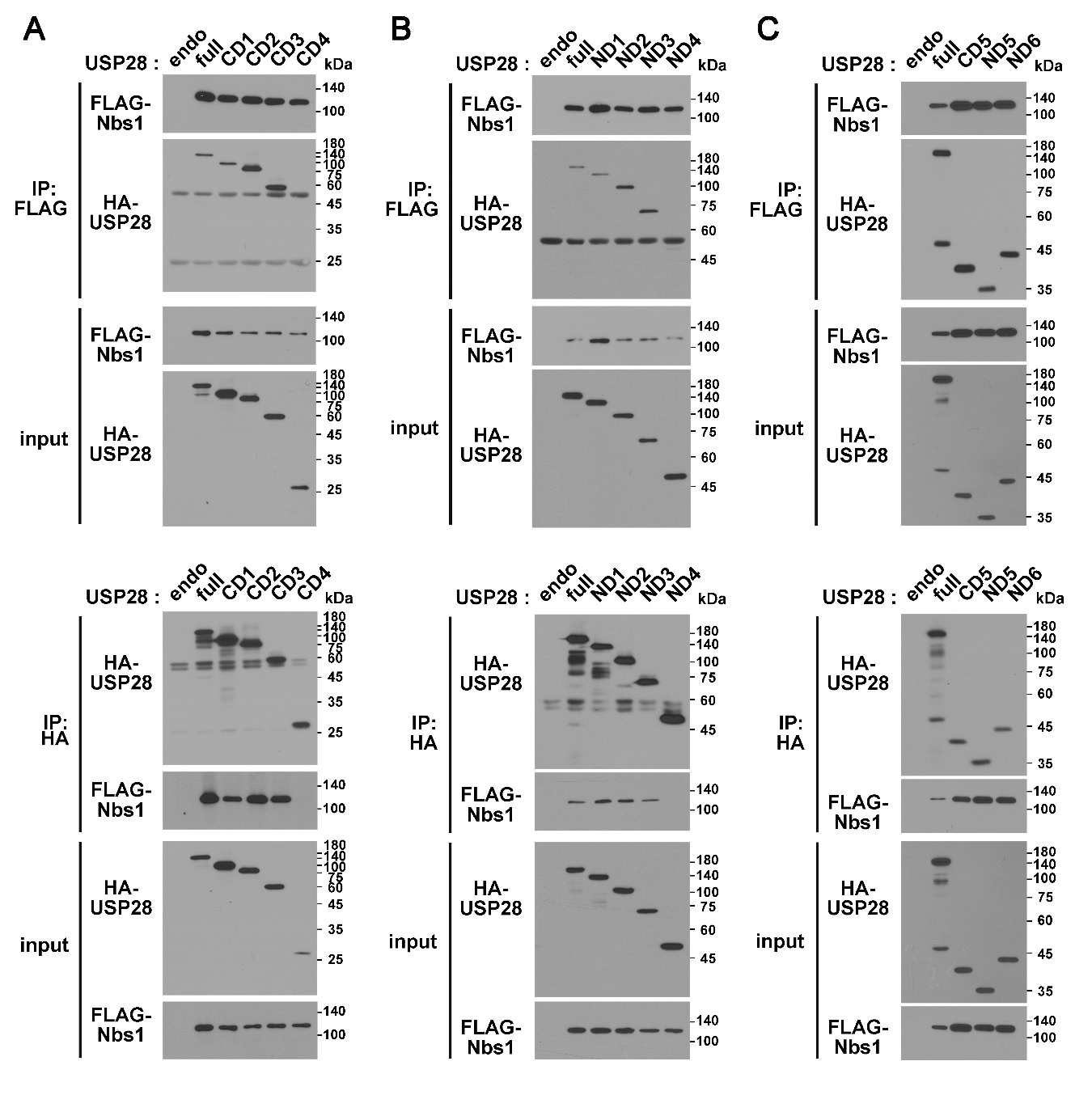


**Figure S6. Immunoprecipitation for mapping the Nbs1-interacting domain in Usp28.** Various truncated HA-Usp28 proteins were expressed with FLAG-Nbs1 proteins in U2OS cells, and immunoprecipitation (IP) was carried out with anti-FLAG-M2-agarose beads (upper panels) or anti-HA antibodies and protein A beads (lower panels). For western blotting of input materials, 10% of extracts used for IP was analyzed with anti-Nbs1 and anti-HA antibodies.


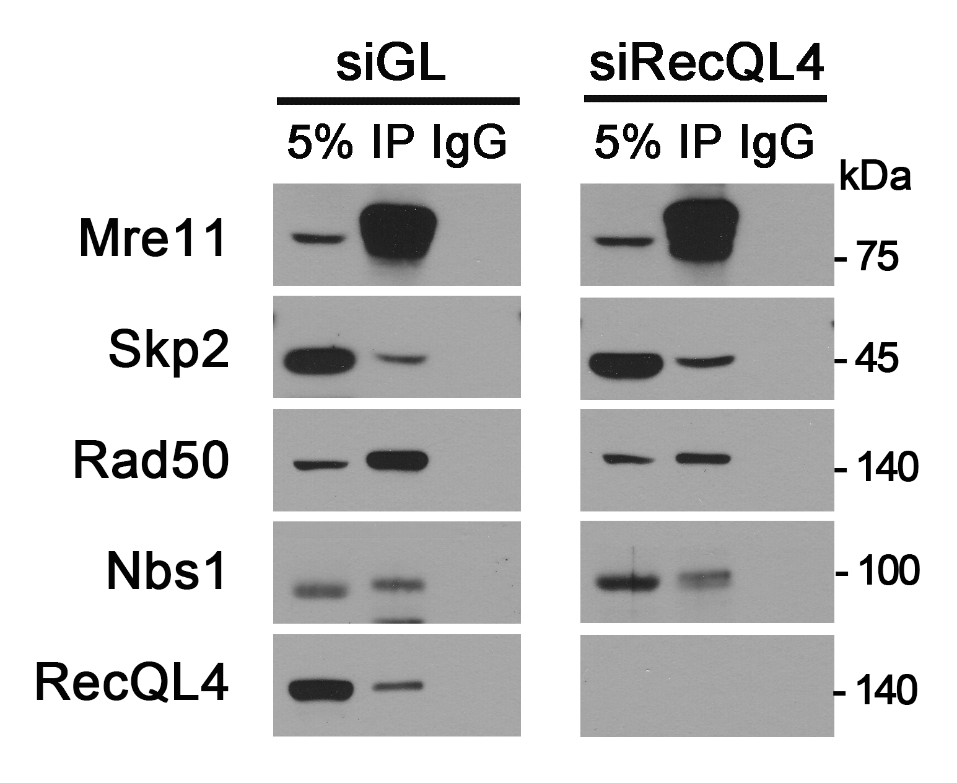


**Figure S7. Skp2 interacts with the Mre11-Rad50-Nbs1 complex regardless of the presence of RecQL4.** RecQL4- or GL2-depleted U2OS cells were pretreated with MG132 (50 µg/mL) for an hour and then treated with neocarzinostatin (NCS; 200 ng/mL) for 15 min, and incubated for 1 h in fresh medium containing MG132. Immunoprecipitation was performed with the anti-Mre11 antibody.


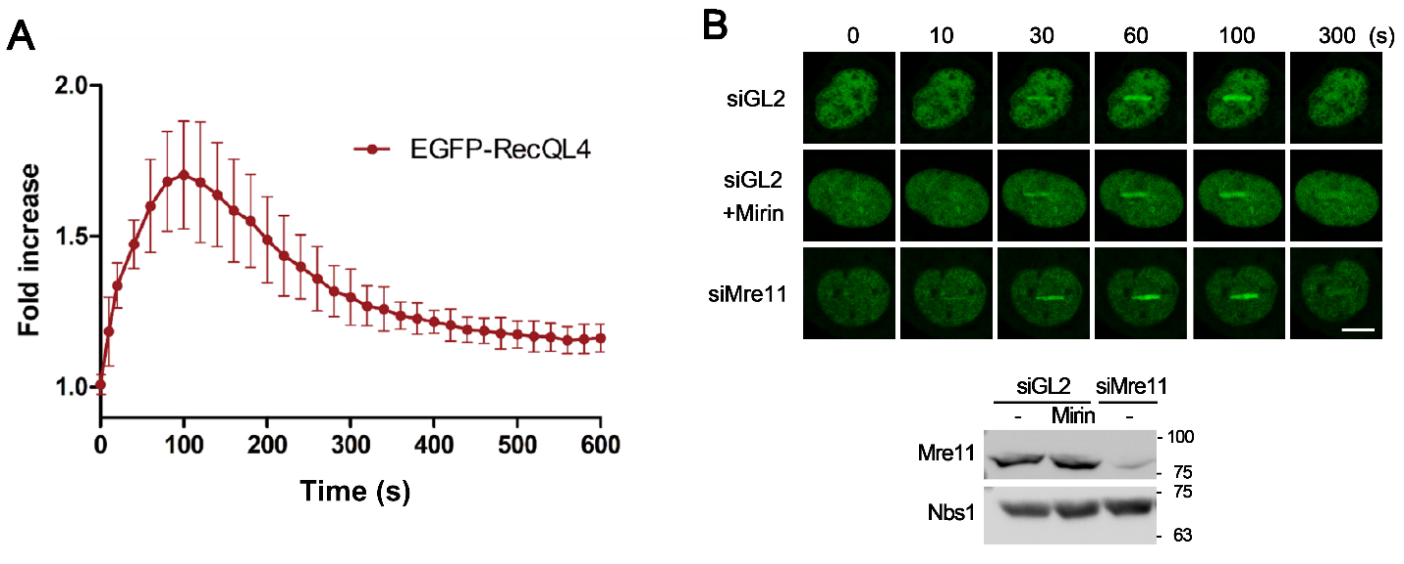


**Figure S8. Recruitment of RecQL4 to the micro-irradiation site is not affected by Mre11 depletion or inhibition of its nuclease activity.** *A,* Association of EGFP-RecQL4 to laser micro-irradiation sites in U2OS cells. Data in graphs are means ± s.d.; n = 15. *B*, Representative images of EGFP-RecQL4 binding to micro-irradiation site in cells treated with siMre11 or mirin, an inhibitor of Mre11 nuclease activity. Lower panel is the result of western blotting showing depletion of Mre11. Scale bar: 10 µm.

**Table S1. Antibodies used in this study**

| Antibody | WB | IP | ICC | Catalog No. |
| --- | --- | --- | --- | --- |
|  | (Dilution) | (Dilution) | (Dilution) |  |
| cyclinA | - | - | 1:100 | sc-271682, Santa Cruz |
| FLAG | 1:3000 | - | - | F1804, Sigma |
| GAPDH | 1:5000 | - | - | sc25778, Santa Cruz |
| HA | 1:2000 | 1:200 | - | AE008, ABclonal |
| K48 | 1:1000 | - | - | ab140601, Abcam |
| lamin B1 | 1:5000 | - | - | ab16048, Abcam |
| Mre11 | 1:3000 | - | 1:500 | GTX30294, GeneTex |
| Nbs1 | 1:1000 | - | 1:100 | A7703, ABclonal |
| pATM(Ser-1981) | 1:1000 | - | 1:200 | #4526, Cell Signaling |
| Rad50 | 1:200 | - | 1:50 | sc-74460, Santa Cruz |
| Rad51 | - | - | 1:100 | GTX100469, GeneTex |
| RecQL4 | 1:1000 | - | - | Prepared in Abfrontier (Korea) (24) |
| RPA32 | - | - | 1:200 | MABE-286, EMD Millipore |
| Skp2 | 1:200 | - | - | sc-7164, Santa Cruz |
| Usp28 | 1:1000 | - | 1:100 | A9292, ABclonal |
| γH2AX | 1:10000 | - | 1:500 | A300-081A, Bethyl |
| γH2AX | - | - | 1:100 | 05-636, EMD Millipore |
| Alexa Fluor 488 anti-mouse IgG | - | - | 1:1000 | A11001, ThermoFisher |
| Alexa Fluor 488 anti-rabbit IgG | - | - | 1:1000 | A11008, ThermoFisher |
| Alexa Fluor 594 anti-mouse IgG | - | - | 1:1000 | A11005, ThermoFisher |
| Alexa Fluor 594 anti-rabbit IgG | - | - | 1:1000 | A11012, ThermoFisher |
